# Wild Norway Rats Do Not Avoid Predator Scents When Collecting Food in a Familiar Habitat: A Field Study

**DOI:** 10.1038/s41598-018-27054-4

**Published:** 2018-06-21

**Authors:** Rafał Stryjek, Berenika Mioduszewska, Ewelina Spaltabaka-Gędek, Grzegorz R. Juszczak

**Affiliations:** 1 Institute of Psychology, Polish Academy of Sciences, Warsaw, Poland; 2 Department of Behavioural Ecology and Evolutionary Genetics, Max Planck Institute for Ornithology, Seewiesen, Germany; 3 Comparative Cognition, Messerli Research Institute, University of Veterinary Medicine Vienna, Medical University of Vienna, University of Vienna, Vienna, Austria; 4 Department of Animal Behavior, Institute of Genetics and Animal Breeding, Jastrzebiec, Poland

## Abstract

The ability to avoid predators is crucial to wild prey animals’ survival. Potential danger is signalled, among others, by the presence of predator scents. These odors are used in research both to trigger and to study fear reactions in laboratory animals; they are also employed as repellents against pest rodent species. In our study, we assessed nine predator-derived odors for their effectiveness in eliciting avoidance responses in a free-living colony of Norway rats (*Rattus norvegicus*). The rats were studied in a field setting. Food was put in two compartments inside the experimental pen: in one of them, predator scent was introduced on experimental days. The rats did not avoid boxes with predator odor and did not display an increased latency of food-carrying behavior or any other fear-related behavior, such as freezing or increased grooming. The results confirm the hypothesis that the foraging of rodents in a well-known territory and in relative proximity to burrows and other shelters is not affected by indirect cues of predation risk, such as the presence of predator urine or feces. We have also concluded that in a well-established colony living in a familiar territory, predator scent holds little promise as rodent repellent.

## Introduction

In the course of evolution, prey animal species have developed specific adaptations which are essential to survival by enabling them to recognize, avoid and defend themselves against predators. Many species are highly sensitive to the presence of predator odors^1^ and reactions to carnivore scents have been observed in many mammal species i.a., Norway rats (*Rattus norvegicus*), brown rats (*Rattus rattus*), house mice (*Mus musculus*), field mice (*Apodemus sylvaticus*), field voles (*Microtus agrestis*), black tailed deer (*Odocoileus hemionus columbianus*), European rabbits (*Oryctolagus cuniculus*), European hedgehogs (*Erinaceus europaeus***)** and sheep (*Ovis ares*)^2^, as well as in birds (house finches, *Carpodacus mexicanus*^3^,). This effect does not seem to result from novelty avoidance, as Holtzman strain of Norway rats (*Rattus norvegicus)* have been observed to respond to a scent from an already present predator (cat, *Felis catus*) and at the same time fail to react to an unexpected odor of aerosol deodorant^4^. Investigations of predator odor avoidance are important because of the value of such data for developing models of anxiety and anxiety disorders^5^, as well as for designing effective pest repellents, which protect crops and plantations^2^ and therefore alleviate human-wildlife conflicts^6^. The popularity of using predator odors as repellents against vermin species is reflected in the wide range of commercially available products developed with the use of predator urine (e.g., PredatorPee Inc.).

According to the predation risk allocation hypothesis^7^, prey species should trade off foraging effort against vigilance (behaviors aimed at predator detection) in relation to the temporal variation of predation risk, i.e., they tend to meet their energy needs by minimizing the risk of being killed. The ratio of risk levels between high- and low-risk situations is assumed to affect the time allocated for foraging, vigilance and other activities. Therefore, the intensity of foraging is predicted to be highest during short periods of low risk interspersed with long periods of high risk and, conversely, to be lowest during short periods of high risk interspersed with long periods of low risk. However, if the exposure to high risk is prolonged, the animals will be forced to forage despite the high-risk circumstances in order to meet their energy intake needs.

Behavioral reactions to predator threat include the inhibition of exploratory activity and non-defensive behaviors (such as grooming, reproduction, foraging, feeding), as well as retreating to lookout locations and change of habitat for a safer one^2,8,9^. A decrease in the number of litter has also been observed in laboratory rats after exposure to predator urine in home areas^10^ and when being housed in close proximity to live predators (Eurasian lynx, *Lynx lynx*;^11^), as well as in wild free-ranging grey-sided voles (*Clethrionomys rufocanus*) after exposure to predator odor^12^.

Although prey species seem to exhibit an innate reaction to odors from sympatric and allopatric predators alike, they might not always recognize certain carnivore odors as threatening^2,13,14^. For example, bush rats (*Rattus fuscipes*) from Australia do not avoid the odor of fox (*Vulpes vulpes*) feces, likely because foxes are introduced predators in the area inhabited by rats^15^. No reaction to feral cat odor was also observed in small marsupials and murids (e.g., yellow-footed antechinus, *Antechinus flavipes*, and ash-grey mouse, *Pseudomys albocinereus*) even though these species have coexisted for more than 200 years^16^. Similarly, the Cypriot mouse (*Mus cypriacus*) did not avoid the odor of its relatively recent main competitor and known predator, the black rat (*Rattus rattus*), whereas it avoided the scent of a domestic cat^17^. It is possible that an aversive response towards predator odors might only occur if the predator and prey share a long evolutionary history and, therefore, the prey becomes genetically programmed to avoid the odors of sympatric predators^18^ (interestingly, a decline in risk-sensitive behavior of prey species seems to be a rapid response to the predator population decline^19^). For these reasons, the negative results obtained in predator odor avoidance studies might reflect the mismatch of predator and prey configurations. However, other studies demonstrate that even certain pairings of prey and predator species without a shared evolutionary history may result in predator odor avoidance^2,20^ and that reduced alien predation pressure after the initial impact might result in the selection for discriminating and avoiding danger in otherwise naïve species^21^.

Typical odor sources are skin, fur, urine, droppings and secretions of the anal glands, such as TMT (2,5-dihydro-2,4,5-trimethylthiazoline) in foxes^2^. Urine is a carrier of many chemical signals in animals. Its constituents such as PEA (2-phenylethylamine^22^) and sulphur compounds (products of digesting animal proteins^23,24^), are present, to varying degrees, in all predators and constitute crucial components generating fear responses in prey species.

Predator odors carry strong effects on the prey animals’ endocrinal systems, by increasing blood pressure and heart rate in rats^25^, as well as by increasing corticosterone level and decreasing testosterone levels in males^2,26^. A significant increase in corticosterone levels has also been observed in Sprague-Dawley rats exposed to TMT, although this effect was not accompanied by defensive behaviors in a small familiar environment^27^. The authors suggested that the observed lack of predator avoidance in a known area may indicate a mechanism which helps the animal react swiftly in case additional stressful stimuli arrive. However, in a study by Storsberg *et al*.^28^, both Lister-Hooded and captive wild rats expressed avoidance behavior in response to TMT, while an upsurge in corticosterone level was observed only in the captive wild rats, which may suggest that the domestication process weakens the physiological reaction to stress in rats. In terms of other physiological reactions, rats exposed to cat odor showed greater Fos immunoreactivity expression in many brain regions than controls, thus suggesting patterns of brain activation in response to stressful stimuli^29^. In addition, rats’ reactions to predator odors provide insights into the innate fear reactions in animals, as well as into the function of the hypothalamic–pituitary–adrenal axis (HPA axis), which coordinates the function of the sympathetic nervous system^22^.

In terms of behavioral reactions to predator odors, experimental data often show diverse relationships. Although predator odor exposure has been proposed as a paradigm for testing both behavioral and neuroendocrine effects, results of some studies have failed to detect both of these changes^30^. In addition, a physiological reaction (an increase in the level of corticosteroids, heart rate and c-Fos immunohistochemistry expression), does not necessarily translate into an avoidance reaction. A study involving 2-propylthietane, the main constituent of weasel (*Mustela nivalis*) anal secretion, suggests a clear distinction between rats’ endocrinal and behavioral responses to predator odors. The results demonstrated significant increases in corticosterone and ACTH (adrenocorticotrophic hormone) levels without any significant accompanying changes in behavioral indices^30^.

Predator odors seem to deter certain prey species and may decrease foraging behavior. However, the effectiveness of such odors remains unclear, as not all studies demonstrate a strong repellent effect. For example, no influence was observed of predator scent on the amount of time spent feeding and on the frequency of alert postures in captive antelopes (Cape grysbok, *Raphicerus melanotis* and gray duiker, *Sylvicapra gimmia*) and on the rates of trapping free-living rodents (*Rhabdomys pumilio* and *Otomys irroratus*) when compared to a control odor^31^. Similarly, in a laboratory study by Bramley and Waas^32^, wild-caught Norway rats demonstrated no avoidance of predator scents in comparison with herbivore scents. In a subsequent field study^32^, forest feeders were marked with synthetic predator odors, but, again, no change was reported in the number or duration of feeder visits by rats, mice (*Mus musculus*) or brushtail possums (*Trichosurus vulpecula*). By contrast, a study conducted on wild-caught Hawaiian roof rats (*Rattus rattus*^33^) in an artificial laboratory setting demonstrated avoidance behaviors in response to predator odors. To conclude, some studies report all predator odors used in the experiments to be ineffective in preventing prey species from approaching odor-marked locations in natural environment, which suggests that further research is necessary before predator odors can be efficiently implemented as commercial pest repellents.

Avoidance reactions, therefore, seem to be modulated by additional external environmental factors (time of day, light intensity, proximity of shelter, familiarity with the territory, etc.^34,35^). In addition, evidence shows that the results of experiments measuring the hormonal correlates of behavior are particularly susceptible to changes in the study subjects’ environment, such as transfer from wild to captive setting or even switch from natural to captive diet^36^.

Moreover, susceptibility to predator odors seems to be modulated by internal factors, such as species characteristics, sex, age, and individual differences^2,21^. A study by Hogg and File^37^ suggests that laboratory-bred rats can be split into two groups: subjects exhibiting innate behavioral responses to predator odor and subjects showing no such reactions. Importantly, these two groups did not differ in their reactions in the presence of neutral odor, in the elevated plus-maze tests of anxiety or in social interactions, but they behaved differently when exposed to a cat scent.

Interestingly, laboratory studies often point to a higher efficacy of certain predator odors than is later reported from field studies in which the same odors are utilized. For example, one of the most frequently investigated odor components, TMT, extracted from the red fox’ feces, was demonstrated to have a strong effect on predator-naïve rats and mice in laboratory studies, but a much weaker effect in field studies^2^. Similarly, in another laboratory study by Sullivan *et al*.^38^, laboratory-housed gophers (*Thomomys talpoides*) avoided TMT but not ferret (*Mustela putorius*), mink (*Mustela vison*) or fox urine-related chemical compounds. The field part of this study, conducted in infested fruit tree orchards, reported no location avoidance effects in any of the odor conditions. However, significantly more gophers were captured in orchards where predator odors were placed in their burrows than were caught before the experiment, which suggests an influence of the presence of predator scent on gopher behavior. Another possibility is that only certain specific behaviors present in wild populations are being inhibited by the presence of predator odors, as in a study by Sparrow *et al*.^39^ where wombats (*Lasiorhinus latifrons*) remained in the vicinity of introduced dingo (*Canis lupus dingo*) scents, but did not dig burrows.

Laboratory experiments, beside their multiple advantages (e.g., full control of the study conditions and considerable control of the distorting variables), are burdened with disadvantages which might explain some of the differences in results obtained in lab and field studies. Experiments involving endocrinology and behavior appear to be particularly susceptible to influences from the environment (natural or laboratory) in which they are performed^36^. The laboratory setting, as an artificial environment, may account for a reinforcement or inhibition of certain behaviors due to the close proximity of humans and to the repeated handling of test subjects^27^. The most important issue in laboratory rats – as products of the domestication process in an artificial environment – is that some adaptive behaviors are to a large extent extinguished (e.g., neophobia, aggression^40–43^). In addition, domestication affects a variety of traits, including morphology, physiology, neural mechanisms and behavior (e.g.,^44–46^).

For these reasons, field studies conducted on wild species, uninfluenced by the domestication process, provide further insights into the mechanisms of animal behavior. Three main behavioral reactions to predator scents have been the focus of such studies: changes in activity patterns, reductions in non-defensive behaviors and habitat shifts^2^. Although field studies fail to allow for a precise control of multiple variables, the ecological validity of such studies is higher than the validity of laboratory studies^47^. Conducting field studies is in line with the ethological principle promoting the study of natural forms of behavior, that is, only such forms of behavior which are of biological significance, such as predator avoidance^48^.

However, available data are inconsistent due to the influences of many potential external and internal factors, such as the predator species used and study setting (laboratory or natural environment). Therefore, the aim of our study was to investigate the capability of nine different kinds of predator odors (sympatric and non-sympatric) to elicit avoidance/fear reaction in a free-living colony of Norway rats. The laboratory strains of the Norway rat are among the most frequently used species in behavioral and biomedical studies^42,49^. The popularity of the species, the vast number of studies conducted so far, as well as the fact that the species is often used as a model for investigations of behavioral and physiological mechanisms and psychic processes in other animal species (including humans), make the rat a particularly interesting study subject. Most studies on predator scent avoidance in rats have been conducted either on laboratory rats^2^ or on wild-caught rats kept in laboratory settings in which they were tested^33,50^, and sometimes even housed, individually^50^. Only some studies investigated fear responses to predator scent in wild rodents in natural free-ranging conditions^31,32,38,51,52^ (for an example of other species see^20^ for a field study on rabbits, *Oryctolagus cuniculus)*. Our study was conducted near the wild rats’ burrows with the aim of investigating predator odor responses in a familiar environment. We hypothesized that rats familiar with the low-risk habitat will not avoid predator scent when collecting food.

## Methods and Materials

### Ethics Statement

Under Polish law, the below-described study did not require permission of the local ethics committee for animal experimentation, as it was a non-invasive experiment based on behavioral observation of free-ranging animals. The study was carried out on private land with permission of its owners. All applied procedures were conducted in accordance with the Polish Animal Protection Act (21 August 1997).

### Animals

The study was conducted on a free-living colony of Norway rats in their natural habitat, on a farm situated on the outskirts of Warsaw, Poland. Based on the observations and data collected from previously conducted studies on this colony^53,54^, the size of the rat population living on the farm had been estimated at approx. 40–50 individuals. The animals were not marked to distinguish between individuals. No pest control had been conducted on the farm for 5 years prior to the experiment – neither mechanically, nor by means of poison. Predators present at the farm which the rat colony had close contact with included cats and dogs (*Canis lupus familiaris*). In addition, the nearby area was home to beech marten (*Martes foina*), weasels, foxes and birds of prey, although these were never encountered on the farm.

### Indoor Pen

**Figure 1:**
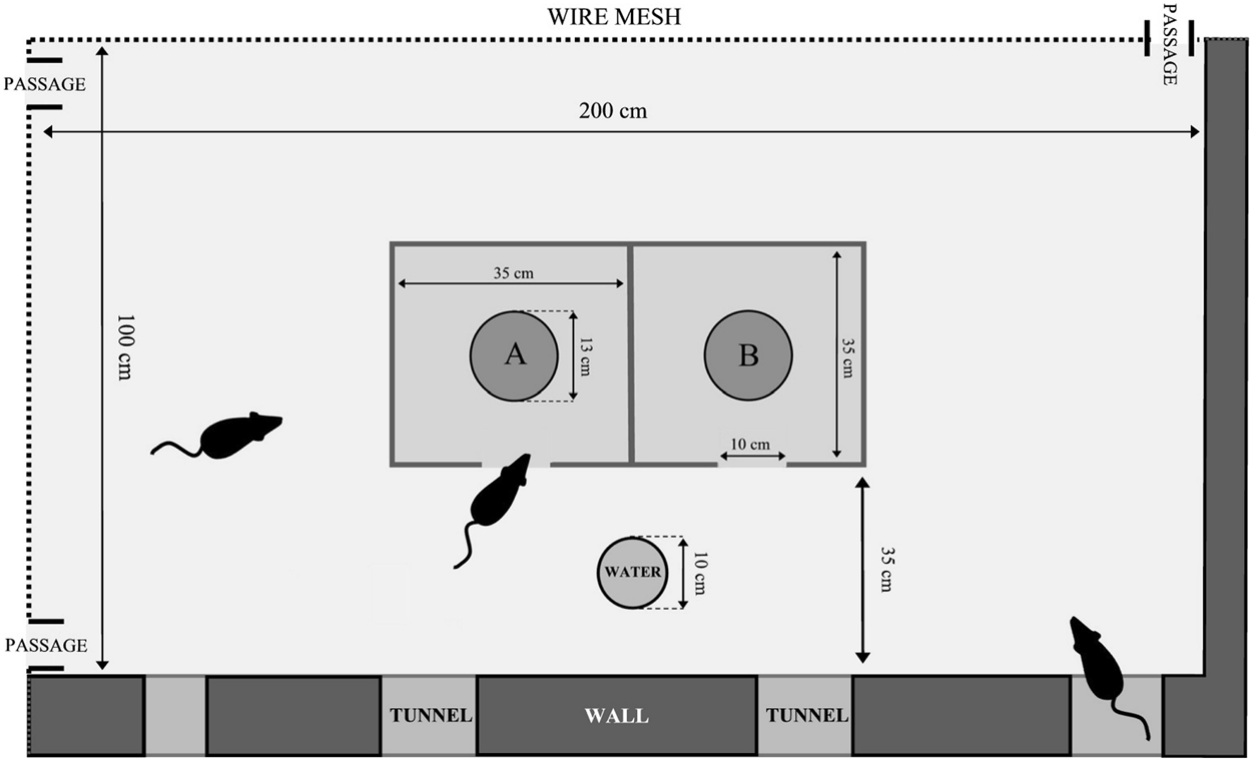
Experimental arena in which the wild rat colony was observed. (**A**,**B**) Flat, rubber bowls, where 15 pellets of laboratory feed were provided at the same time of day on a daily basis. During experimental sessions, cardboard pieces at the bottom of bowl A or bowl B were soaked with predator urine.

The rats were observed in a barn located on the farm, in a dog- and cat-proof indoor pen fenced with a wire mesh (200 cm/100 cm/100 cm; see Fig. 1). Dog and cat proofing was implemented to prevent predator-induced variability of rat behavior^8^. We used 4 infrared cameras connected to a digital video recorder, which enabled 24/7 motion detection recording. The rats could easily access the pen through 5 to 8 entrances – the number of entrances (of which 3 to 4 were entrances leading directly from the rats’ underground burrows to the pen) varied in time, as rats typically modify their burrow entrance placement. The nests were situated in the adjacent room. The maximum light intensity in the box and in the surrounding space was 5–25 lx and 10–50 lx, respectively, depending on whether the barn gate was shut or open.

To minimize the risk of influence of human and animal scents, neither the researchers nor other vertebrate animals entered the pen during the study. Food bowls, as well as feed and water, were supplied and put on the ground through a cover placed on top of the pen. Water was provided ad libitum. The staff used disposable nitrile gloves at all times. Due to mice infestation of the barn, which could influence the results of the experiment (both by changing the rats’ behavior and due to food intake by mice), the size of the mouse colony had been reduced significantly by placing mouse live traps in the building one week prior to the start of the experiment. No signs of mice presence in the indoor pen were observed during the experiment.

### Apparatus

A box built from OSB (Oriented Strand Board), with external dimensions of 74 × 37.5 cm and wall height of 40 cm (see Fig. 1 and Video 1), was placed at the center of the indoor pen. The box was comprised of two independent compartments with single entrances (10 × 10 cm) located on the same wall of the box. A black rubber bowl (13 cm in diameter and 1.5 cm high) was placed in each of the compartments (A and B – Fig. 1). Pieces of black cardboard were fixed to the bottoms of the bowls by a double-sided adhesive tape; the cardboard pieces were soaked with predator scent (during experimental sessions) or with water (in the control compartment). The bottom of the boxes was covered with earth, which was identical to the ground surface in the remaining part of the experimental arena (clayish sand); the earth layer was ca. 10 mm thick.

### Odor Probes

Samples of predator urine: foxes, coyotes (*Canis latrans*) and mountain lions (*Puma concolor*), were purchased from PredatorPee Inc. (Bangor, ME, USA). Urine of female Sumatran tigers (*Panthera tigris sumatrae*) and urine of African lions (*Panthera leo*) were collected at the Warsaw ZOO by means of a purpose-built sewer system that enabled urine collection from a slanted concrete floor. Lion feces was obtained from the Warsaw ZOO. Urine samples from healthy unneutered male cats and dogs were collected by a local veterinarian by means of bladder catheterization. TMT samples were purchased from Contech Enterprises Inc. (Victoria, Canada). The set of urine samples included odors of both sympatric (cat, dog, fox) and allopatric species (mountain lion, African lion, Sumatran tiger, coyote). In the study, fresh urine samples (i.e., applied several hours after collection and not frozen; lion, cat, dog) were used alongside frozen urine samples (mountain lion, coyote, fox).

### Procedure

The procedure applied in the experiment resembled the experimental scheme successfully applied in one of the studies conducted by our team on the same rat colony^54^ and the observational method used to determine changes in subjects’ behavior after exposure to predator scents is considered an appropriate behavioral test of olfaction^55^.

The experiment took place in winter 2015/2016 (from 20 January until 18 March – 59 days in total). The average daily temperature in the pen was 6.3 °C (min. −4 °C, max. 10 °C) and the average humidity level was 69% (min. 65%, max. 72%). The temperature during the experimental days was always above 5 °C. The times of sunrises ranged from 8:35 a.m. at the start to 6:44 a.m. at the end of the experiment, whereas the times of sunsets ranged from 5:00 p.m. to 6:46 p.m.

The rats were well habituated to standard laboratory fodder pellets (Labofeed H, WP Morawski, Kcynia, Poland), as well as to the time of feeding - three non-invasive studies^53,54^ on food and object neophobia had been conducted over the period of twelve months prior to the experiment. The animals were fed at approx. 3 p.m., i.e., before the peak of their circadian activity, which falls immediately after twilight^43^. To habituate the animals to the experimental procedure, during the first 7 days of the experiment, 15 food pellets (ca. 20 mm long, 12 mm in diameter, 3.5 g +/−0.5 g in weight) were put in the bowls (A and B), located in the two separate compartments of the experimental box (see Fig. 1). Following the habituation period, for 3 subsequent days, the food (15 standard pellets) was served in one of the compartments, in the bowl with the cardboard bottom soaked on a daily basis with predator odor immediately before feeding. Urine samples (1 ml) and lion feces (1 ml of water solution) were smeared on the cardboard bottom with a brush. TMT scent was introduced by fixing 3 10 µl pipette tips, to the bowl by means of an odorless glue; the pipette tips had been used to apply TMT in another study (Storsberg *et al*.^28^) and they were subsequently stored in an airtight box, which prevented the disappearance of the scent. Three plastic pieces of similar size were fixed to the bowls in the control box. Fifteen food pellets were also put in the adjacent compartment, which served as control measurement. The procedure with the new predator odor was repeated for 3–5 consecutive days. To prevent habituation to continuous novelty, every experimental period was followed by 2 days during which the animals were given the standard feed in both compartments. To avoid the effect of place, the bowls with the new predator odor were supplied alternately in the right-hand and left-hand compartments. After the period of introducing a given odor was over, to eradicate all traces of smell, the earth from the bottom of the box was replaced in both compartments and new bowls were put in.

After the experiment, three individuals from the colony were captured and tested for *Toxoplasma gondii*, which may modulate host behavior, decreasing the level of fear of predator odor^56,57^.

### Statistical Analysis

The following variables were calculated based on the video-recordings: the latency to pick individual pellets (calculated from the moment the first rat entered the specific compartment) and the number of instances where rats approached the food without picking the pellet (calculated until the last of the 15 pellets was picked). Only behaviors that were recorded during periods from the moment the pellets were supplied until 8 a.m. on the following day were taken into consideration. The methodological inconvenience of not having individuals marked was partly offset by the number of measurements performed (latency to pick 30 food pellets, over a period of 59 days), by the considerable size of the colony and by a qualitative analysis.

As in a study conducted previously^54^, two points of reference for the reaction to the introduced novelty (in this case: predator odor) were used: cross-sectional (comparison of the rats’ behavior in the compartment with predator scent and in the compartment with standard bowl and unscented fodder) and longitudinal (analysis of the animals’ behavior when exposed to predator odor at subsequent stages of the experiment). See Video 1 to obtain a general view of the rats’ behavior in the experimental setting.

## Results

**Figure 2:**
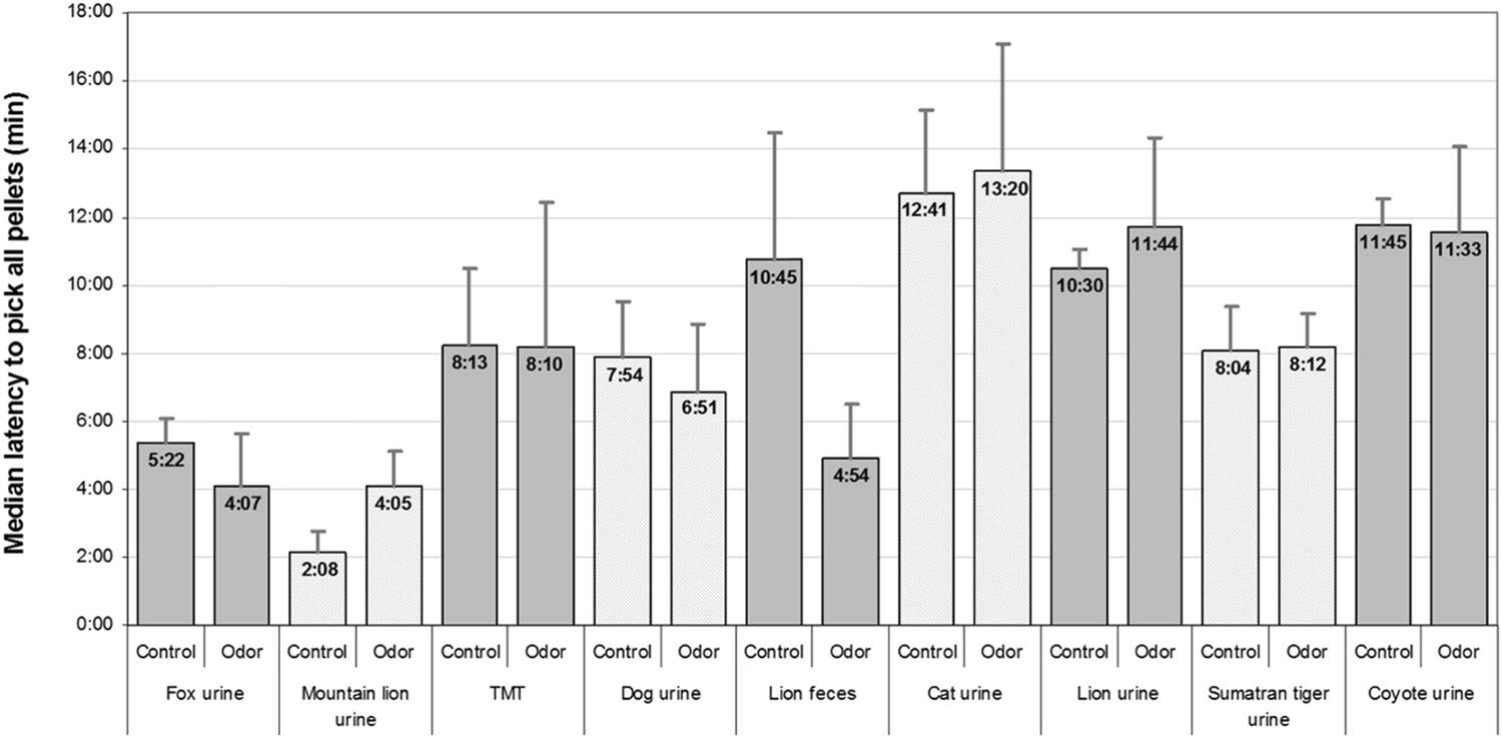
Median latency to pick all pellets for control and experimental compartments in response to exposure to various predator odors. Error bars represent SM (standard error of the mean).

**Figure 3:**
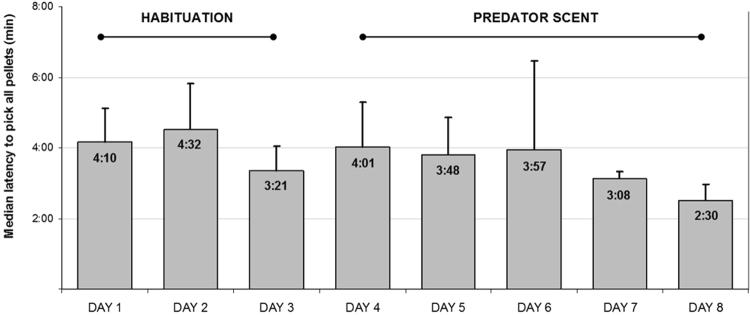
Median values of latency (min) to pick all 15 pellets prior (habituation sessions) and after introducing predator scent in the experimental compartment (experimental sessions). Error bars represent SM (standard error of the mean).

None of the introduced odors induced avoidance behavior in the rat colony under study (see Figs 2 and 3 and Table 1). The maximum latency to eat the last of the 15 pellets was only 26 minutes 12 seconds in the experimental sessions, and 57 minutes 3 seconds in the initial habituation sessions. A more detailed data analysis was conducted using non-parametric statistical tests. Differences were considered significant for *P* values of < 0.05.

**Table 1:** Median latencies (min) of food-carrying in experimental and control compartment depending on odor source.

Species		Odor probe	Experimental compartment		Control compartment	
			Latency of taking 1 pellet	Total time of taking all pellets	Latency of taking 1 pellet	Total time of taking all pellets
Fox	*Vulpes vulpes*	urine	0:01	4:07	0:01	5:22
Mountain lion	*Puma concolor*	urine	0:02	4:05	0:02	2:08
Fox	*Vulpes vulpes*	TMT (anal gland secretion)	0:01	8:10	0:01	8:13
Dog	*Canis lupus familiaris*	urine	0:02	6:51	0:01	7:54
Lion	*Panthera leo*	feces	0:01	4:54	0:01	10:45
Cat	*Felis catus*	urine	0:01	13:20	0:01	12:41
Lion	*Panthera leo*	urine	0:02	11:44	0:02	10:30
Sumatrian tiger	*Panthera tigris sumatrae*	urine	0:02	8:12	0:01	8:04
Coyote	*Canis latrans*	urine	0:01	11:33	0:01	11:45

### Cross-sectional Analysis

The statistical analysis commenced with a comparative examination of the rats’ behavior in the experimental compartment (where predator odor was introduced) and in the control compartment, where the cardboard bottom of the bowl was soaked with water instead of predator scent (see Fig. 2 and Table 1). No differences were observed in the behavior of rats between compartments with regard to the pace at which pellets were picked, measured as the length of time that elapsed between instances of picking individual pellets (Mann-Whitney *U* test – *U* = 605, *P* = 0.93). The number of instances where pellets were approached but none of them were picked did not differ either (*U* = 521, *P* = 0.113); there was no difference in the latency to pick the first pellet (*U* = 531.5, *P* = 0.268), the last pellet (*U* = 561, *P* = 0.545) and the number of instances of entering the compartments after picking all the pellets, which served as an exploration indicator (*U* = 529.5, *P* = 0.328).

A Kruskal-Wallis test was carried out to evaluate the differences among the nine predator odors with respect to the median latency to pick all food pellets (see Fig. 2). The test revealed no differences [*χ*2(8, N = 35) = 12.97, *P* = 0.113].

To assure the robustness of the obtained results, additional tests were undertaken. Firstly, mean latency of picking all food pellets were regressed on a dummy variable taking value of 1 for the experimental compartment and 0 for the control compartment. Under t (0.648), F (0.420) and Wald (0.420) test null hypothesis of equal means cannot be rejected at any conventional level. Secondly, Bayesian Zellner regression^58^ with the same variable was performed. Regardless of the choice of the g prior – Unit Information Prior^59^ and Risk Information Criterion^60^ – the hypothesis of equal means was maintained. The obtained point estimates with standard errors in parentheses under aforementioned prior structure are 0.692 (1.083) are 0.351 (0.773) respectively. This confirms the results obtained with the use of a nonparametric test.

### Longitudinal Analysis

Next, we carried out a comparison of the animals’ behavior when confronted with predator odor at subsequent stages of the experiment (see Fig. 3). The factor taken into consideration was the rats’ reaction in the initial phase of novel odor provision (the first 3–5 days during which the novel scent was supplied). The reaction was compared to the rats’ behavior in the preceding 3-day period when no novel scent was provided and when the animals were given the standard (unscented) pellets in both compartments.

No differences at the subsequent stages of the experiment were observed with regard to any of the variables. The median latency to pick first food pellet was not a time-varying parameter [the Friedman test value was *χ*2(7, N = 4) = 12.303, *P* = 0.09], nor was the latency to pick the last pellet [*χ*2(7, N = 4) = 2.95, *P* = 0.95], or the number of instances of entering the pen without taking the food [*χ*2(7, N = 4) = 3.71, *P* = 0.81].

Similarly to the case of cross-sectional data to assure the robustness of the obtained results, additional test were undertaken. Firstly, the latency of picking the first pellet, the latency of picking the last pellet, and the number of instances of entering the pen without picking the food were all regressed on a dummy variable taking value of 0 for habituation days and 1 for experimental days. Secondly, Bayesian Zellner regression^58^ with the same variables was performed, and with the use of the two different g priors described previously. In all the circumstances, the hypothesis of equal means could not be rejected.

None of the rats consumed the food on the spot. The food which had been picked by rats from the box compartments was always taken away from the box and carried into nearby tunnels. The box was entered by individuals of different ages (assessed by body size) and of both sexes (both males and females were trapped in the pen after the experiment for parasite screening). No regular activity pattern was observed which correlated with the age/size of animals entering the pen. No behavioral signs of stress (i.e., grooming and freezing) or exploration (prolonged sniffing, rearing, unusual locomotor activity) were observed during the tests. The *Toxoplasma gondii* test conducted on 3 individuals captured after the completion of the study produced a negative result.

## Discussion

None of the predator odors used elicited avoidance behavior in the colony of wild rats. Furthermore, rats did not differ in their foraging latency either in the test (predator scented) or in the control compartment; there were no differences in the intensity of exploration of these two locations (entering the pen without collecting food). No difference between the nine predator odors used in the study was detected with respect to foraging, and there were no differences, at the subsequent stages of the experiment, in latency to start foraging and in the intensity of exploration.

The results obtained follow a literature trend where field studies on predator odor responses demonstrate lesser scent effects than laboratory studies^2,61,62^. Such results indicate that avoidance behavior is not automatic and does not rely on direct cues of predator presence, such as urine odor, but it is rather mediated by various environmental factors, such as type of habitat, temperature and weather^34,35,61,63^, and social context^64,65^.

The barn rats were exposed to predator scents in a familiar and safe environment (which enabled them to move along walls and ensured the proximity of burrows and other shelters), which is indicated by the observed lack of behavioral signs of stress (i.e., grooming and freezing) or exploration of the pen. An alternative explanation of the lack of grooming behavior may relate to a possible tendency of animals to minimize the time spent in the enclosure, and thus to restrain from displaying behaviors other than locomotion and food carrying. No predators entered the experimental arena and, therefore, the experimental situation might have been perceived by rats as connected to low risk, despite the presence of predator scent. Similarly, in a study by Koivisto and Pusenius^66^, the presence of a predator (weasel) caused a rapid decrease in foraging behavior in voles, whereas exposure to predator scent only had a weaker effect. The authors suggested that odor indicates a recent predator presence in a location; however, as weasels frequently move from one place to another, the risk in scent-marked areas might be perceived by voles as not very high. In addition, the costs of movement and thermoregulation in the pen were low, since the tunnel cover/escape routes were close by and foraging occurred indoors during winter (temperatures on experimental days were always above 5 °C). The relatively small distance to cover from the location at which an animal forages decreases the energetic cost of foraging (via low costs of movement and thermoregulation) and, likewise, reduces the risk of predation^67^. No avoidance behavior was observed (measured either qualitatively or quantitatively), as the anti-predator effort in low-risk circumstances drops to low levels^7^. Furthermore, the low level of vigilance observed (no sniffing, no freezing) resulted in a higher energetic gain^67^.

Most laboratory studies on rats and mice use one of the unconditioned tests of anxiety (the plus maze, the light-dark box or the open field test); these paradigms, however, should be approached with caution with regard to the possible interpretations of behavioral data collected^68^. Aspects worth considering include the questionable measures of anxiety (subjects might exhibit neophobic reactions or natural preferences for enclosed spaces rather than anxiety^69^) and the intra-individual variation in behavioral responses to anxiety, both of which might diminish the reliability of test measures^70^. In addition, in an open unfamiliar space, two stress-inducing factors are present, namely the experimental predator odor and a perceived higher risk of predation. Various habitat structures provide different degrees of predation risk and, therefore, modify the spatial behavior of prey species in response to odors^71^. Such an effect was demonstrated in a study by Cohen *et al*.^72^, which showed that exposure to cat odor increased fear responses in rats placed in the elevated plus-maze: they were less likely to enter the open (but not closed) arms of the maze. Another explanation might be the fact that standard experimental protocols used in laboratory tests include animals exposed to long-term low-risk conditions and short-time predator-related stressors during testing^7^, which are likely to result in an overestimation of the intensity of anti-predator behavior expected in field situations, where perceived long-term predation risk might be considered higher than in a laboratory setting.

Another ecological factor which contrasts our field study with laboratory tests is the social context: subjects lived and were tested in their natural environment and in their social group instead of being tested individually, as is usually the case in a laboratory setting. Rats are highly social animals living in colonies^42^, which dilutes the predation threat^64,65^. Moreover, Norway rats have a relatively small home range, and, consequently, when forced to change their habitat, it is unlikely that they will explore an unfamiliar area far away from the nest, which is often the case in laboratory settings^73^. Although foraging with other group members involves the cost of lower food intake per individual^74^, benefits include higher food sources detection, lowered predation risk due to the dilution effect^64^ and an overall increased group vigilance^75^. Therefore, on the one hand, rats in the company of other colony members might be more likely to forage and explore despite the presence of predator odors. On the other hand, a group setting includes potential opportunities for food sharing/scrounging without the need of some colony members to enter a potentially high-threat area. None of the rats in our study consumed the pellets within the pen, but they took them into the tunnels. Such behavior opens the possibility for food sharing or food theft (either tolerated or non-tolerated) of pellets collected by the pen dwelling individuals^76^.

Alternative interpretations of the results include a possibility of a parasite infection, methodological aspects, habituation to predator odors, and individual differences among rats.

*Toxoplasma gondii* causes an infection which might change the behavior of its host by decreasing fear reactions to predator odors^56,57^. However, medical examination of three members of the barn colony produced a negative test result and, therefore, absence of avoidance reactions to predator scents observed in the barn was not a result of a compromised health of the rat colony.

Multiple aspects of study design can potentially influence the resulting repellent effect of predator odor. Some possible elements include: the incorrect context of predator scent presentation^63,77^, the use of different sizes of odor-scented stimuli (cloths^78^), or the age^79^ and form^2,80^ of the predator stimulus used. The odor source (either fur, urine, feces or anal glands) influences its effectiveness in eliciting avoidance behavior, with fur seemingly causing the most significant endocrinal and behavioral changes. One possible explanation of this effect is that predator fur may be a stronger signal of predatory threat/presence than feces or urine^81^. However, fur-derived odors are difficult to control in an experimental situation, as it is unclear which active ingredient in fur gives rise to its potent repellent properties^2^. Additionally, the molecular complexity of the odor signal is also important, with single compounds being generally less effective in eliciting defensive reactions in prey than odor arrays. Such arrays consist of different volatile compounds which may convey more relevant information about the predation risk, e.g., what prey species are consumed by a given predator^80^. Age of the predator odor was also linked to its repellent effect, as presented in a study by Hegab *et al*.^79^ where Brandt’s voles (*Lasiopodomys brandtii*) exhibited more defensive responses to freshly defrosted cat feces than to cat feces stored between 2 and 8 days, and in a study by Bytheway *et al*.^51^ where free-ranging rats showed a decrease in defensive behavior in response to aged predator odor. Another possibility is that interactions mediated via olfactory cues between predators and prey are more complex than previously assumed^82^. Instead of consisting of dyadic interactions between a single predator (signaller) and prey (receiver), a complex olfactory web of information may be created by visitations of scent patches by multiple species from various levels of the food chain.

Linked to study design is a possibility that rapid habituation to predator odors may be also a factor limiting their repellent strength^2,62^. For example, habituation to the repellent effects of cat odor has been observed in laboratory rat studies^83^. Although rats exhibit an increase in bloodstream corticosterone after being presented with a cloth that had been rubbed on a cat, habituation of this endocrinal response occurs after repeated exposure to the odor stimulus^84^. However, in our study, habituation to predator scent was unlikely to explain the lack of avoidance reaction, as no changes in subjects’ behavior were observed at the subsequent experimental stages, and our experiment involved 2-day inter-session breaks after each testing session, when the rats were given the standard non-scented pellets in both test compartments.

On an individual level, only very bold animals might have been entering the experimental arena and, therefore, no scent avoidance was observed. In addition, individual differences in pre-experience with predators also modify the behavior of prey species^2^. In our study, all the rats in the barn colony had had similar exposure to dogs, cats, and humans before the onset of the experiment and, therefore, no large individual variance in terms of pre-experience occurred in the test sample. However, as our study did not aim at investigating the influence of individual differences on antipredator behavior, further studies are needed to provide insights into the link between personality and reactions to predator odors.

Some limitations in the presented study should be acknowledged. Firstly, the exact sample size and composition are unknown due to the absence of individual marking. However, the size of the whole colony was estimated at approx. 40–50 individuals in previous studies^53,54^ and two complementary kinds of statistical approaches were applied to assure the robustness of the acquired results - both classical and Bayesian analysis showed a strong support for the null hypotheses (no influence of predator scent on the observed rats’ behavior). Additionally, indirect data suggests that the sample included subjects of various ages (estimated by body size) and both sexes (males and females were trapped in the experimental area after the study for parasite analysis). Secondly, the study was conducted on a single wild rat colony which limits the extent of general conclusions. Nevertheless, the investigated context was of high practical value as a cohabitation of rats and humans on a single farm presents ample opportunities for human-wildlife conflicts to occur. Therefore, analysis of the behavior of the whole rat colony, beside shedding a light upon general aspects of behavioral reaction to predator odor in free-living rats, provides valuable insights for the development of pest repellent techniques.

The results of the study and areas for future research can be concisely summarized by quoting Stoddart^85^: “Clearly they do not always react to the odor of their predators, but do they ever?”. Potential modifications of future studies investigating behavioral aspects of predator odor avoidance in wild rats can be categorized according to two methodological aspects, namely study design and predator odors. Firstly, to gain more insights into predator avoidance behavior in various natural circumstances, experiments could be conducted in habitats with a perceived higher predation risk (such as outdoor areas, plantation fields, and/or areas further away from tunnels and burrows). Secondly, the predator scents utilized could be obtained from different species and from various organic source material. A study by Arnould and Signoret^86^ suggests that in domestic sheep (*Ovis ares*), unusual odors from non-sympatric species (lion feces) had a smaller repellent effect than dog feces (however, other studies have supported the use of lion feces as a repellent against deer, *Cervus elaphus*^87,88^). A subsequent study^89^ also included wolf (*Canis lupus*) feces, which, together with dog feces, produced the strongest repellent effect. Other organic materials include fur and skin, as predator odors obtained from these body areas were suggested to have a more profound effect on prey species than odors derived from urine or feces^2^. There are also indications that such odors may have longer-lasting effects, as cat fur/skin scent is a highly aversive stimulus and triggers context conditioning when used as an unconditioned stimulus, while other types of predator odors (derived from feces, urine and anal gland secretions) apparently do not. Another direction for future studies is to include a biochemical analysis in order to gain a fuller understanding of wild rats’ reactions to predator odors. A study on Sprague-Dawley rats^27^ reported that TMT-induced biochemical effects may occur without the expression of defensive behaviors depending on the rats’ environment (safe vs risky).

The high variability of reactions of rat populations and individual animals observed in field studies is a factor that decreases the effectiveness of predator odors as pest management tools. Avoidance is not unconditioned, but it is rather mediated by various environmental factors and rats’ high behavioral plasticity^49^, which facilitates the changeability of their foraging strategies. The results of our study confirm the hypothesis that foraging of rodents in a well-known territory and in a relative proximity to burrows and other shelters is not affected by indirect cues of predation risk, such as the presence of predator urine or feces. Lack of response to the odors used in the study suggests that animals rely more on the habitat characteristics, such as i.a. closeness of shelter and light intensity. We also conclude that predator scent in the case of a well-established colony in a familiar territory holds little promise as a rodent repellent. Despite methodological limitations of the presented study, it provides valuable insights and directions for future studies, which might involve investigations of odor influence in unknown and, therefore, higher risk areas. Reactions to predator odors seem to depend on a variety of variables, such as odor source, intensity, context of exposure, in addition to the inter-specific variety of prey species and individual characteristics^2^. Further studies are necessary to shed more light on this complex network of interlinked factors.

## Electronic supplementary material

video 1
